# Using the GHQ-12 to screen for mental health problems among primary care patients: psychometrics and practical considerations

**DOI:** 10.1186/s13033-020-00397-0

**Published:** 2020-08-10

**Authors:** S. G. Anjara, C. Bonetto, T. Van Bortel, C. Brayne

**Affiliations:** 1 Cambridge Institute of Public Health, University of Cambridge, School of Clinical Medicine, Cambridge Biomedical Campus, Forvie Site, Robinson Way, Box 113, Cambridge, CB2 0SR UK; 2 Department of Neurosciences, Biomedicine and Movement Sciences, University of Verona, Piazzale L.A. Scuro 10, 37134 Verona, Italy

**Keywords:** Mental health, Primary care, Screening, Psychometrics, Indonesia, Low- and Middle-Income Countries, Receiver Operating Curve, Confirmatory Factor Analysis

## Abstract

**Background:**

This study explores the factor structure of the Indonesian version of the GHQ-12 based on several theoretical perspectives and determines the threshold for optimum sensitivity and specificity. Through a focus group discussion, we evaluate the practicality of the GHQ-12 as a screening tool for mental health problems among adult primary care patients in Indonesia.

**Methods:**

This is a prospective study exploring the construct validity, criterion validity and reliability of the GHQ-12, conducted with 676 primary care patients attending 28 primary care clinics randomised for participation in the study. Participants’ GHQ-12 scores were compared with their psychiatric diagnosis based on face-to-face clinical interviews with GPs using the CIS-R. Exploratory and Confirmatory Factor Analyses determined the construct validity of the GHQ-12 in this population. The appropriate threshold score of the GHQ-12 as a screening tool in primary care was determined using the receiver operating curve. Prior to data collection, a focus group discussion was held with research assistants who piloted the screening procedure, GPs, and a psychiatrist, to evaluate the practicality of embedding screening within the routine clinic procedures.

**Results:**

Of all primary care patients attending the clinics during the recruitment period, 26.7% agreed to participate (676/2532 consecutive patients approached). Their median age was 46 (range 18–82 years); 67% were women. The median GHQ-12 score for our primary care sample was 2, with an interquartile range of 4. The internal consistency of the GHQ-12 was good (Cronbach’s α = 0.76). Four factor structures were fitted on the data. The GHQ-12 was found to best fit a one-dimensional model, when response bias is taken into consideration. Results from the ROC curve indicated that the GHQ-12 is ‘fairly accurate’ when discriminating primary care patients with indication of mental disorders from those without, with average AUC of 0.78. The optimal threshold of the GHQ-12 was either 1/2 or 2/3 point depending on the intended utility, with a Positive Predictive Value of 0.68 to 0.73 respectively. The screening procedure was successfully embedded into routine patient flow in the 28 clinics.

**Conclusions:**

The Indonesian version of the GHQ-12 could be used to screen primary care patients at high risk of mental disorders although with significant false positives if reasonable sensitivity is to be achieved. While it involves additional administrative burden, screening may help identify future users of mental health services in primary care that the country is currently expanding.

## Background

In 2015, Indonesia had only 773 psychiatrists for 250 million residents [1]. This shortage of specialist mental health professionals is shared by most Low- and Middle-Income Countries (LMICs). This is reflected in the treatment gap and low proportion of people who receive adequate mental health care for their needs. While the median worldwide Treatment Gap for psychosis is 32.2% [2], the treatment gap in Indonesia is more than 90% [3]. Mental health problems are estimated to be present in around 20–36% of patients attending primary care settings and when untreated, result in significant suffering and growing healthcare costs [4, 5]. Improving ways to identify people at risk of mental health problems is a feasible strategy to help bridge the Treatment Gap and reduce their suffering [6].

Embedding a screening procedure into primary care could help early identification, intervention, and prevention of common mental disorders, including anxiety and depression [7]. Screening scales allow for a more systematic assessment of self-reported mental health problems. For a screening procedure to be effective, a reliable screening instrument is necessary, and its optimal threshold needs to be determined. Screening alone cannot and will not improve the outcomes for common mental disorders such as depression, if resources for effective intervention must also be in place [8]. In Indonesia, mental health services are increasingly provided at zero or very low costs in primary care following the systematic introduction of the World Health Organization (WHO) Mental Health Gap Action Programme to 10,000 primary care clinics [9].

The General Health Questionnaire (GHQ) is a self-administered screening tool designed to detect current state mental disturbances and disorders in primary care setting [10]. The GHQ has been translated into 38 languages since its development, indicating its face validity across cultures [11]. While the GHQ was originally developed as a 60-item questionnaire, several abridged versions (30-item, 28-item, 20-item, and 12-item) are currently available. The 12-item version was adopted as a screening tool in a multi-country World Health Organization (WHO) study of mental disorders in primary care setting, as it was considered the best validated among similar inventories [12–14].

The twelve-item General Health Questionnaire (GHQ-12) is intended to screen for general (non-psychotic) mental health problems among primary care patients [12]. Items on the GHQ-12 are rated on a 4-point scale using a timeframe of “in the last two weeks.” There are three ways of scoring the GHQ-12: the bimodal GHQ scoring method (0-0-1-1) recommended by the test authors for use in clinical settings; and the Likert scoring method (0-1-2-3) which is commonly used in research, and the C-GHQ scoring method where positively phrased items are scored (0-0-1-1) and negatively phrased items (0-1-1-1).

A review of international validity studies of GHQ-12 conducted 20 years ago, including in LMICs, reported that the optimal threshold varied from 1/2 to 6/7, with the most common cut-off being 2/3 [12]. Considering 17 more international studies revealed a range of thresholds from 0/1 to 5/6 [15]. Table 1 shows later studies, and their distribution of thresholds [4, 7, 16–36]. These differences may be the result of varying prevalence rates of mental disorders and comorbidity, as well as the populations in which the scale was administered and cultural influences [37].

**Table 1:** A Sample of GHQ-12 Threshold Studies on Various Clinical Populations after 1998

Author	Country	Sample	Threshold	Scoring
Hardy [22]	England	Health care employees	3/4	GHQ-12
Kuruvilla [27]	India	Primary care patients	2/3	GHQ-12
Bhui [17]	England	Punjabi primary care patients	2/3	GHQ-12
Aydin [16]	Turkey	Tubercolosis and chronic obstructive pulmonary disease patients	3/4 Tubercolosis 5/6 COPD	GHQ-12
Cano [18]	USA	Veterans	1/2	GHQ-12
Daradkeh [20]	UAE	Undergraduates	15/16	Likert
Donath [21]	Australia	General population	3/4 0/1	C-GHQ GHQ-12
Makowska [29]	Poland	Working adults	2/3	GHQ-12
Holi [23]	Finland	General population	3/4	GHQ-12
McKenzie [31]	Australia	Gulf war veterans	1/2	GHQ-12
Picardi [33]	Italy	Dermatology patients	3/4	GHQ-12
Martin [30]	UK	Individuals with facial disfigurement	0/1	C-GHQ and GHQ-12
Navarro [32]	Spain	Postnatal women	4/5	GHQ-12
Schmitz [13]	Germany	Primary care patients	11/12	Likert
Shelton [35]	England	Postnatal women	4/5 14/15	GHQ-12 Likert
Krespi Boothby [26]	Turkey	Breast cancer patients	1/2 affective disorders 1/2 generalised anxiety disorder 3/4 major depression	GHQ-12
Yusoff [36]	Malaysia	Medical students	3/4	GHQ-12
Baksheev [7]	Australia	High school students	9/10 for males 10/11 for females	Likert
Caraveo-Anduaga [19]	Mexico	Primary care patients	2/3	GHQ-12
Cornelius (2013)	The Netherlands	Disability claimants	19/20	Likert
John [24]	India	General population	3/4	GHQ-12
Kim [25]	Korea	General population	2/3	GHQ-12
Lundin [28]	Sweden	General population	11/12 5/6 1/2	Likert C-GHQ GHQ-12
Ruiz [34]	Colombia	Undergraduates; female adults; psychiatric patients	2/3 11/12	GHQ-12 Likert

The first GHQ-12 validity and reliability study in Indonesia was published in 2006, where GHQ-12 was compared against Symptom Checklist (SCL-90) as the gold standard, in a community-based prevalence study [38]. A Confirmatory Factor Analysis (CFA) found the Indonesian version of the instrument to have two factors: psychological distress and social dysfunction. Since then, the Indonesian language version of the GHQ-12 has been extensively used in numerous research studies.

A more recent study examined the validity of the GHQ-12 as a screening tool for Adjustment Disorder in Indonesian primary care setting [39]. This study shows that the GHQ-12 is valid and reliable for use with adjustment disorder, Cronbach’s α = 0.863 for Likert scoring and 0.841 for bimodal scoring. For Adjustment Disorder, sensitivity and specificity for GHQ-12 were.81 and 0.62 (for the optimum cut-off point ≥ 11 in Likert scoring method), 0.81 and 0.57 (for the optimum cut-off point ≥ 2 in bimodal scoring method). The study further conducted CFAs of the different scoring methods, each finding agreement with different existing theoretical models.

This study aims to examine the psychometrics and practicality of using GHQ-12 to screen for common mental health problems among Indonesian adult primary care patients. The feasibility of the screening procedure will be evaluated by embedding it into routine patient flow for 2 weeks in a pilot study, followed by a focus group discussion with stakeholders involved in the implementation. Cronbach’s alpha will indicate the scale’s internal consistency. CFAs will be used to determine construct validity as used in previous studies [40]. Receiver Operating Characteristic (ROC) curves have been widely used to describe and compare the performance of diagnostic algorithms [41] and will be used to determine the most appropriate threshold score.

## Methods

### Context

There are approximately 10,000 state-owned primary care clinics in Indonesia, providing free access to medical and dental care for residents of each clinic’s catchment area. These clinics, called *Puskesmas*, also provide care at a nominal fee for non-residents. This study recruited participants from 28 *Puskesmas* in Yogyakarta, Indonesia, as part of a pre-study of a cluster randomised controlled trial [9]. These 28 *Puskesmas* provide mental health services. All *Puskesmas* in the province have received ISO accreditation standardising their patient flow and administrative procedures, making it possible to embed a uniform screening procedure across the clinics.

### Design

This is a cross sectional study conducted to test the validity and screening accuracy of the GHQ-12 and determine the point at which the balance between sensitivity and specificity is optimised. This study piloted the recruitment procedures for a trial examining the clinical and cost-effectiveness of two mental health care frameworks for primary care [9]. A pilot study was conducted in June 2016 to test the screening procedure.

### Ethics

Ethics approval for the study and larger trial was granted by the University of Cambridge Psychology Research Ethics Committee (reference number PRE.2015.108) and Universitas Gadjah Mada (reference number 1237/SD/PL.03.07/IV/2016). Trial insurance further covers investigators and research participants (University of Cambridge Trial Insurance reference number 609/M/C/1510). Permission to conduct research at the Province of Yogyakarta including its all five districts was obtained from the Provincial Government Office (reference number 070/REG/V/625/5/2016). Additional permits were also obtained from each of the five districts. Ethics approval from individual clinics (*Puskesmas*) were not required as all clinics are funded and managed by district governments. The trial which this study was embedded in has been registered with clinicaltrials.gov since 25 February 2016, NCT02700490.

### Participants

Participants were primary care attendees recruited over a period of 2 weeks in December 2016. These patients present with physical ailments at the adult general care clinic of the *Puskesmas*. Patients pick up a queue number and a GHQ-12 form, which they self-completed while waiting for routine blood pressure checks. Patients were then invited to take part in the study regardless of their GHQ-12 score. From 2532 consecutive primary care patients who completed the GHQ-12, 26.7% (676) consented to additional in-depth psychiatric interview. The interviews were conducted by a general medical practitioner (GP) blinded to their patients’ GHQ-12 score.

### Measures

#### General Health Questionnaire (GHQ-12)

The primary measure being assessed for its screening accuracy is the Bahasa Indonesia version of the GHQ-12. Prior to patient recruitment, the lead author (SGA) reviewed the items with the 28 clinicians from participating sites to ensure content and semantic validity. The same version had been used in previous validation studies with various clinical populations. In the Bahasa Indonesia version, items 2, 5, 6, 9, 10, and 11 are negatively phrased. This study took place in ‘real life’ clinical setting, suggesting the appropriateness of the bimodal scoring method (0-0-1-1). As this study aims to examine the adequacy of the GHQ-12 as a screening tool, lifetime diagnoses were not taken into consideration. Instead, current mental health status was evaluated.

#### Clinical Interview Schedule-Revised (CIS-R)

For the evaluation of mental health, GPs used the Clinical Interview Schedule-Revised (CIS-R) [42], following the protocol of similar validity studies in Italy, England, Brazil, and Chile [15]. The CIS-R [42] is a fully structured diagnostic instrument that was developed from an existing instrument, the Clinical Interview Schedule (CIS), designed to be used by clinically experienced interviewers [43]. The CIS was revised and developed into a fully structured interview to increase standardisation and to make it suitable to be used by trained lay interviewers in assessing minor psychiatric morbidity in the community, general hospital, occupational and primary care research. As the CIS-R specifically diagnoses mood and anxiety disorders, participants with indication of other disorders (psychosis, sleep disorders, dementia) were asked additional questions which enabled the interviewers to establish an ICD-10 diagnosis.

For our sample, interviews were conducted by GPs. The psychiatric diagnostic criteria of the ICD-10 are widely used in the Indonesian health system as the Indonesian manual for diagnosing psychiatric disorders (*Pedoman Panduan Diagnosa Gangguan Jiwa*) released in 1993 and used by medical doctors and psychologists, was a translation and adaptation of the ICD-10 released by the WHO in 1992.

#### Data analysis

IBM SPSS version 24.0 and IBM SPSS Amos version 24.0 were used to conduct the Confirmatory Factor Analysis (CFA) and ROC. Exploratory factor analysis (EFA) was first conducted with the same dataset, to explore whether the data would replicate either the one, two, or three-factor solutions previously reported. The EFA yielded a three-factor solution, which we have labelled distress, anxiety, and social function. This model was further tested in the subsequent CFA. Consistent with previous EFA analysis, the principal components method was used, with orthogonal (Varimax) rotation. Following the EFA, four models were tested for goodness of fit (CFA):

1. Three-dimensional: as indicated by the EFA, the GHQ-12 was modelled as a measure of three latent variables (distress, anxiety, and social function).
2. One-dimensional: the GHQ-12 was modelled as a measure of one construct (psychiatric morbidity) using all 12 items. The model indicates one latent variable with twelve indicator variables, each with its own error term.
3. Two-dimensional: the GHQ-12 was modelled as a measure of two latent variables (psychological distress and social dysfunction) as found in a previous validation study in Indonesia [38]. The model indicates items 2, 5, 6, 9, 10, and 11 correspond to psychological distress, while the rest correspond to social dysfunction.
4. One-dimensional with correlated errors: the GHQ-12 was modelled as a measure of one construct but with correlated error terms on the negatively phrased items, modelling response bias [44]. This model is identical to model 2, but with correlations specified between the error terms on the negatively phrased items.

Following the CFA, a ROC analysis was conducted. The required sample size for a prospective ROC study of a single diagnostic test [45] allowing a type I error of 0.05 and a power of 0.80, with the more conservative AUC1 of 0.80, AUC0 of 0.70, and the allocation ratio of 4 (prevalence of common psychiatric disorders is estimated to be 20% in the primary care population, thus the prevalence of non-diseased is estimated at 80%) was 370 subjects (74 clinically confirmed cases and 296 clinically confirmed non-cases).

The ROC curve analysis is a commonly used method for visualising performance ability and grouping classification [46]. The ROC analysis plots a test’s true positive rate (sensitivity) against its false positive rate (1-speficity) [47]. The area under a ROC curve represents the probability that a randomly chosen subject is correctly rated or ranked with greater suspicion than a non-diseased subject [48]. The area under the curve (AUC) ranges from 0.5 for models with no discrimination ability, to 1 for models with perfect discrimination ability [49]. A ROC curve that is near the point of perfect classification (upper left corner of the ROC space) is considered superior for detection performance [50].

In addition, the positive predictive value (PPV) describes the proportion of all positive results that are correct; while the negative predictive value (NPV) describes the proportion of all negative results that are correct. These predictive values are dependent on the prevalence of mental disorders in the study sample [51].

Total GHQ-12 scores were utilised as the test variable for the ROC analysis. The gold standard against which the GHQ-12 was tested was the presence of diagnosis following an in-depth psychiatric interview using the CIS-R. Two-by-two contingency tables were created by cross-tabulating diagnostic outcomes (the presence or absence of any mental disorders) and the GHQ-12 screening outcomes (positive or negative screening on the GHQ-12).

#### Pilot study and focus group discussion

The pilot study was conducted over a period of 1 week in June 2016. Trained and vetted research asistants checked in for duty every morning at 7 a.m. A tally of the number of screenings completed was checked against *Puskesmas* attendance at the end of every day, which enabled the calculation of the percentage of adult primary care attendees screened. In total, 5341 patients were screened within the pilot period.

At the end of the pilot, stakeholders who were involved in the screening process and a psychiatrist (expert in cultural psychiatry) were invited to participate in a focus group discussion (FGD) to discuss the challenges of implementing the screening procedure, scoring, operational burden, and informing patients of the outcomes. In total, six GPs and research assistants participated in the FGD, which took place in September 2016. The FGD was semi-structured and explored the following topics:

- Primary care patients’ comprehension of the screening questionnaire;
- Feasibility of the screening procedure according to the flow of patients in the clinics;
- Common issues encountered during the screening process;
- General feedback about providing mental health services in primary care.

As two GPs declined to have the FGD recorded, a researcher was taking notes during the FGD process. The notes were discussed with other co-authors and analysed for the purpose of ensuring the feasibility of the screening process.

During the FGD, it became clear that while the screening procedure largely worked, older patients required help with reading the screening questionnaire. Patients picked up the screening questionnaire alongside a queue number at the registration counter, filled the questionnaire while waiting for routine blood pressure check (all adult patients are required to pass through the blood pressure counter). A staff nurse checking patients’ blood pressure could assess the screening questionnaire visually as the GHQ scoring method (0-0-1-1) required no advanced arithmetic. The clinics generally had difficulty keeping their pens as patients accidentally took them home. It was evident that GPs required between 20 and 60 min more with each patient who screened positive, creating a long queue in the waiting rooms. GPs reported that as they get used to asking patients about their mental health symptoms, the additional interviews could become quicker. When patients were asked to return for an in-depth psychiatric interview at a later date, unfortunately most did not return.

## Results

### Sample characteristics

Participants were aged between 18 and 82 years old (median 46). From the 2532 primary care patients approached, 676 consented to participate (452 women; 224 men). Median and interquartile range for women were 2 and 4, and for men 2 and 3. The difference in median scores between women and men was not significant (Mann–Whitney U = 47,981.50, p = 0.253).

The table below presents participants’ demographic characteristics (age, marital status, education level), as well as their GHQ-12 scores by gender.(Table 2).

**Table 2:** Total and by gender socio-demographic characteristics and GHQ-12 scores (0-0-1-1 scoring)

	Women (N = 452)		Men (N = 224)		Total (N = 676)	
	N	%	N	%	N	%
Age (11 missing)						
18–29	117	26.2	47	21.4	164	24.7
30–39	62	13.9	24	11.0	86	12.9
40–49	118	26.5	28	12.8	146	22.0
50–64	119	26.7	81	37.0	200	30.0
65+	30	6.7	39	17.8	69	10.4
Marital status (2 missing)						
Unmarried	77	17.1	54	24.1	131	19.4
Married	319	71.1	163	72.8	482	71.7
Separated/Divorced/Widowed	53	11.8	7	3.1	60	8.9
Education (6 missing)						
Elementary	94	21.0	33	14.9	127	19.0
Middle school	104	23.2	43	19.4	147	21.9
High school	157	35.0	97	43.6	254	37.9
Diploma	20	4.5	16	7.2	36	5.4
University	48	10.7	27	12.2	75	11.2
Others	25	5.6	6	2.7	31	4.6
GHQ-12 Score						
Median (IQR)	2.00 (4.00)		2.00 (3.00)		2.00 (4.00)	

Almost one in five (19%) had only completed elementary-level education. A further 21% completed Junior High School, and 37.9% completed a high school diploma. The rest (22.1%) completed undergraduate or postgraduate degrees. Fewer than 5% received less than 6 years of formal education.

Table 3 shows the prevalence of ICD-10 psychiatric diagnoses and GHQ-12 median scores for adult Indonesian primary care patients. For those with a severe depressive episode, the GHQ-12 median score was 10, with an interquartile range of 7. For those with Comorbid Anxiety and Depression, the GHQ-12 median score was 3, with an interquartile range of 3. For those with general anxiety disorder the GHQ-12 median score was 6, with an interquartile range of 9.

**Table 3:** Total and by gender prevalence of psychiatric diagnoses and median GHQ-12 scores (bimodal scoring) of respondents interviewed with CIS-R and further clinical interviews

ICD-10 diagnoses	Men	Women	Total	GHQ-12
	N (%)	N (%)	N (%)	Median (IQR)
Mild depressive episode	7 (1.0)	29 (4.3)	36 (5.3)	2 (3)
Moderate depressive episode	1 (0.1)	11 (1.6)	12 (1.8)	7 (4)
Severe depressive episode	1 (0.1)	4 (0.6)	5 (0.7)	10 (7)
Mixed anxiety and depression	31 (4.6)	71 (10.5)	102 (15.1)	3 (3)
General anxiety disorder	7 (1.0)	18 (2.7)	25 (3.7)	6 (2)
Panic disorder	5 (0.7)	15 (2.2)	20 (3.0)	5 (6)
Social phobia	7 (1.0)	13 (1.0)	20 (3.0)	2 (1)
Agoraphobia	1 (0.1)	0 (0.0)	1 (0.1)	2 (0)
Specific isolated phobia	2 (0.3)	6 (0.9)	8 (1.2)	3.5 (2)
Obsessive compulsive disorder	0 (0.0)	3 (0.4)	3 (0.4)	5 (0)
Diagnosis of other disorders	15 (2.2)	54 (8.0)	69 (10.2)	2 (3)

Median scores for those with a diagnosis (cases) compared to those who do not meet the ICD-10 diagnostic criteria (non-cases) are shown in Table 4.

**Table 4:** GHQ-12 mean and median scores for non-cases vs. cases meeting any ICD-10 diagnostic criteria during sampling period, Bimodal scoring (0-0-1-1)

	Women		Men		All	
		N		N		N
Mean (SD)						
Cases	3.70 (2.66)	235	3.61 (2.64)	89	3.68 (2.65)	324
Non-cases	1.40 (1.79)	216	1.21 (1.58)	135	1.33 (1.71)	351
Median (IQR)						
Cases	3 (3)	235	3 (3)	89	3 (3)	324
Non-cases	1 (2)	216	1 (2)	135	1 (2)	351

The GHQ-12 median for cases (48%) was 3, with an interquartile range of 3, and the median for non-cases was 1, with an interquartile range of 2. The group meeting diagnostic criteria had significantly higher median scores than those without diagnosis (Mood’s Median Test χ^2^ = 111.07, df = 1, p < 0.001).

### Reliability

The Cronbach’s alpha of the GHQ-12 for bimodal scoring (0-0-1-1) was 0.76, indicating satisfactory internal consistency. Inter-rater reliability was not applicable as the GHQ-12 was self-completed by patients. Test–retest reliability was not conducted for this study.

### Factor analyses

Table 5 shows the Pearson correlation coefficient for all items. EFA (principal components analysis with Varimax rotation) suggested a three-factor solution explaining 48.0% of the total variance in items (factor 1 eigenvalue = 3.4, factor 2 eigenvalue = 1.3, and factor 3 eigenvalue = 1.1). We label the factors distress, anxiety, and social function.

**Table 5:** Pearson Correlation Matrix between all items

	GHQ2	GHQ3	GHQ4	GHQ5	GHQ6	GHQ7	GHQ8	GHQ9	GHQ10	GHQ11	GHQ12
GHQ1	0.242	0.194	0.185	0.231	0.175	0.184	0.198	0.127	0.256	0.104	0.180
GHQ2	1.000	0.087	0.115	0.185	0.162	0.193	0.219	0.167	0.178	0.124	0.165
GHQ3		1.000	0.266	0.066	0.187	0.217	0.179	0.117	0.177	0.118	0.158
GHQ4			1.000	0.094	0.152	0.197	0.306	0.101	0.232	0.140	0.193
GHQ5				1.000	0.308	0.162	0.217	0.363	0.239	0.231	0.288
GHQ6					1.000	0.158	0.466	0.302	0.356	0.271	0.248
GHQ7						1.000	0.264	0.213	0.122	0.081	0.201
GHQ8							1.000	0.216	0.255	0.223	0.265
GHQ9								1.000	0.331	0.300	0.446
GHQ10									1.000	0.496	0.293
GHQ11										1.000	0.287
GHQ12											1.000

Table 6 shows the rotated component matrix for all items.

**Table 6:** Rotated component matrix for exploratory factor analysis

	Factor 1	Factor 2	Factor 3
GHQ1	0.11	*0.54*	0.26
GHQ2	0.11	*0.66*	0.02
GHQ3	0.06	0.12	*0.68*
GHQ4	0.11	0.10	*0.72*
GHQ5	*0.50*	0.47	− 0.18
GHQ6	*0.55*	0.20	0.26
GHQ7	0.03	*0.58*	0.31
GHQ8	0.34	0.32	*0.45*
GHQ9	*0.66*	0.31	− 0.11
GHQ10	*0.70*	− 0.02	0.29
GHQ11	*0.75*	− 0.16	0.16
GHQ12	*0.57*	0.30	0.06

Extraction Method: Principal Component Analysis (Items loading on the factos are indicated in *Italics*.)

Rotation Method: Varimax with Kaiser Normalization

Rotation converged in 7 iterations

**Fig. 1:**
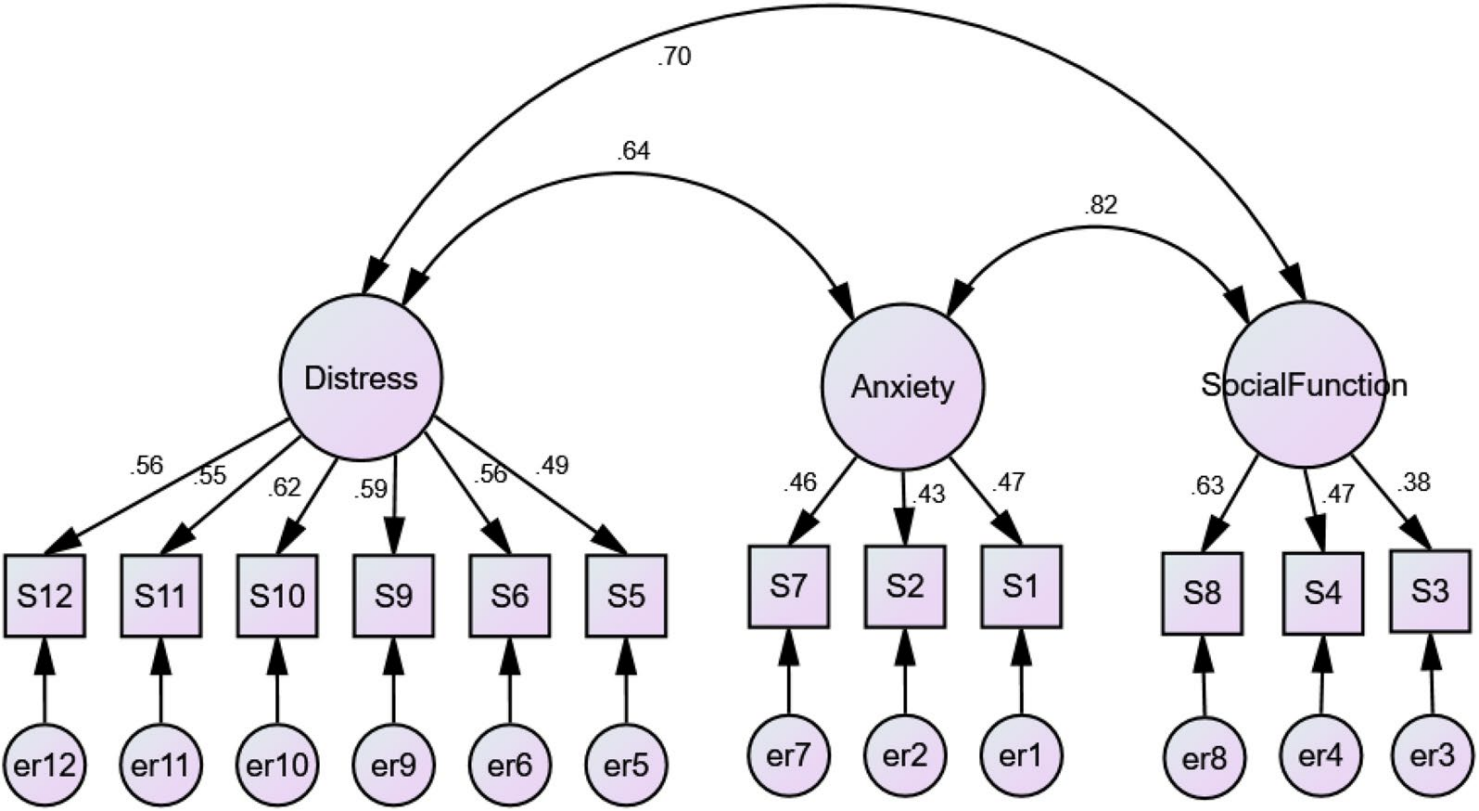
Confirmatory Factor Analysis of three-factor model

**Fig. 2:**
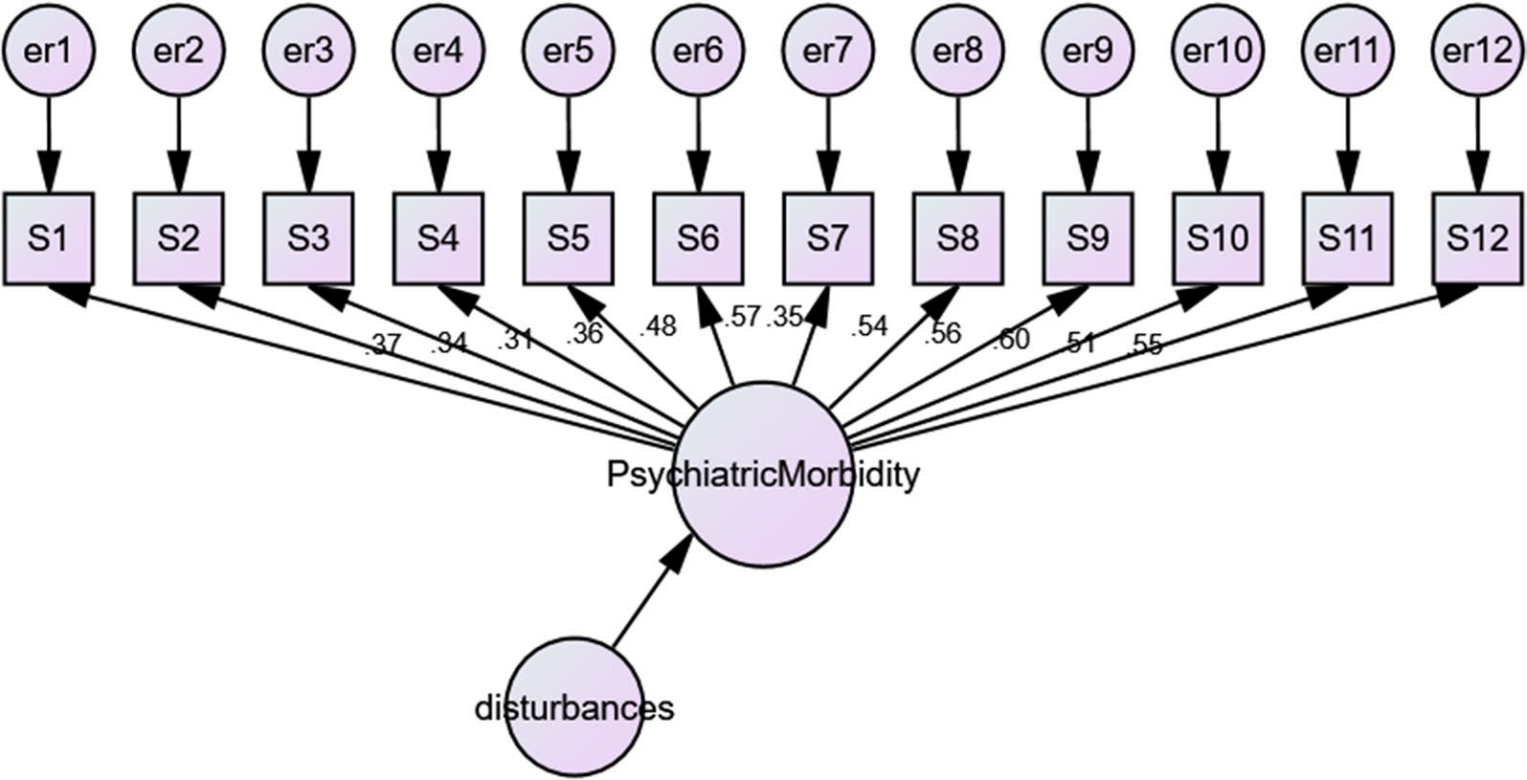
Confirmatory Factor Analysis of a one-dimensional model

**Fig. 3:**
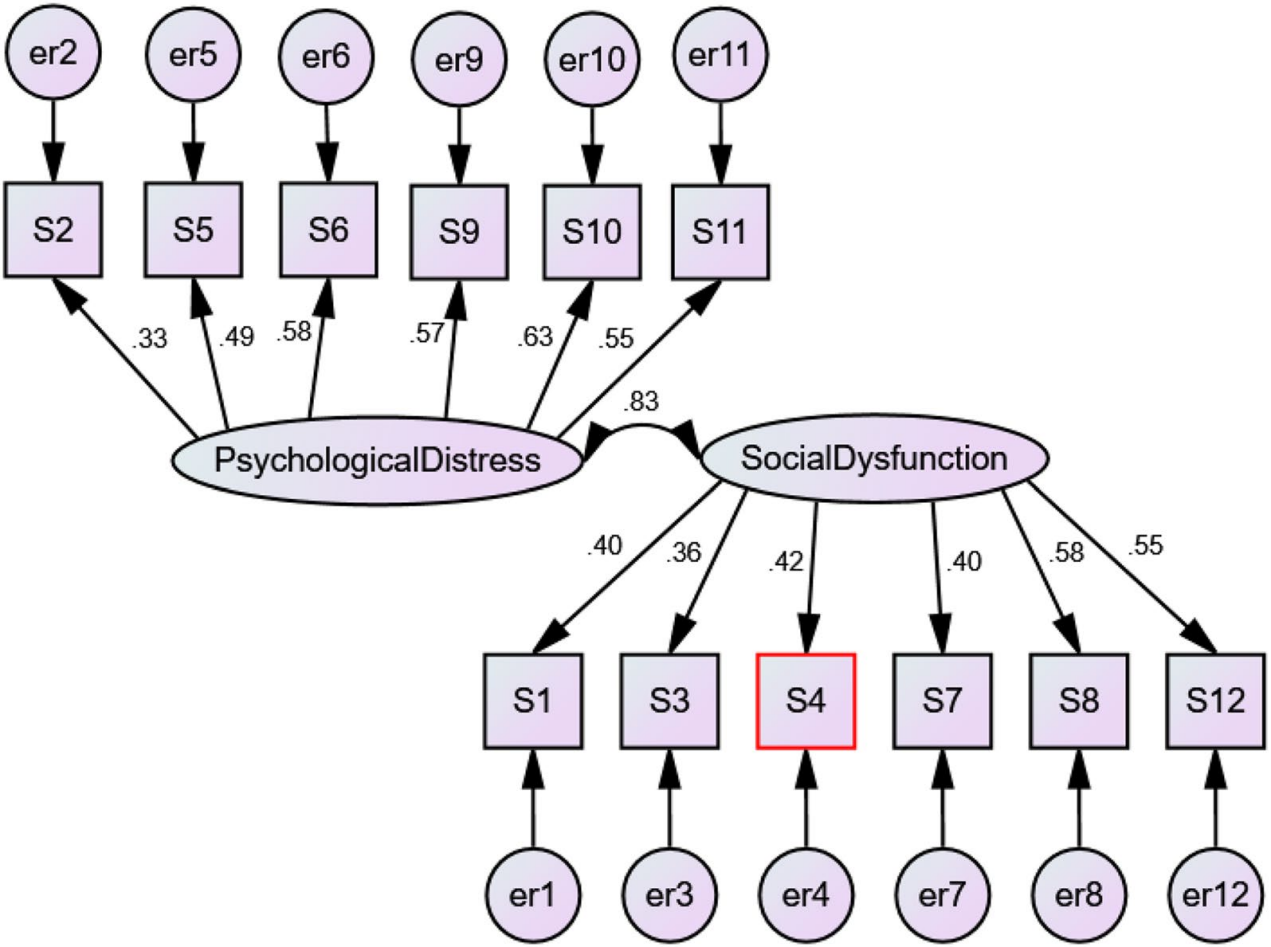
Confirmatory Factor Analysis of a two-dimensional model

**Fig. 4:**
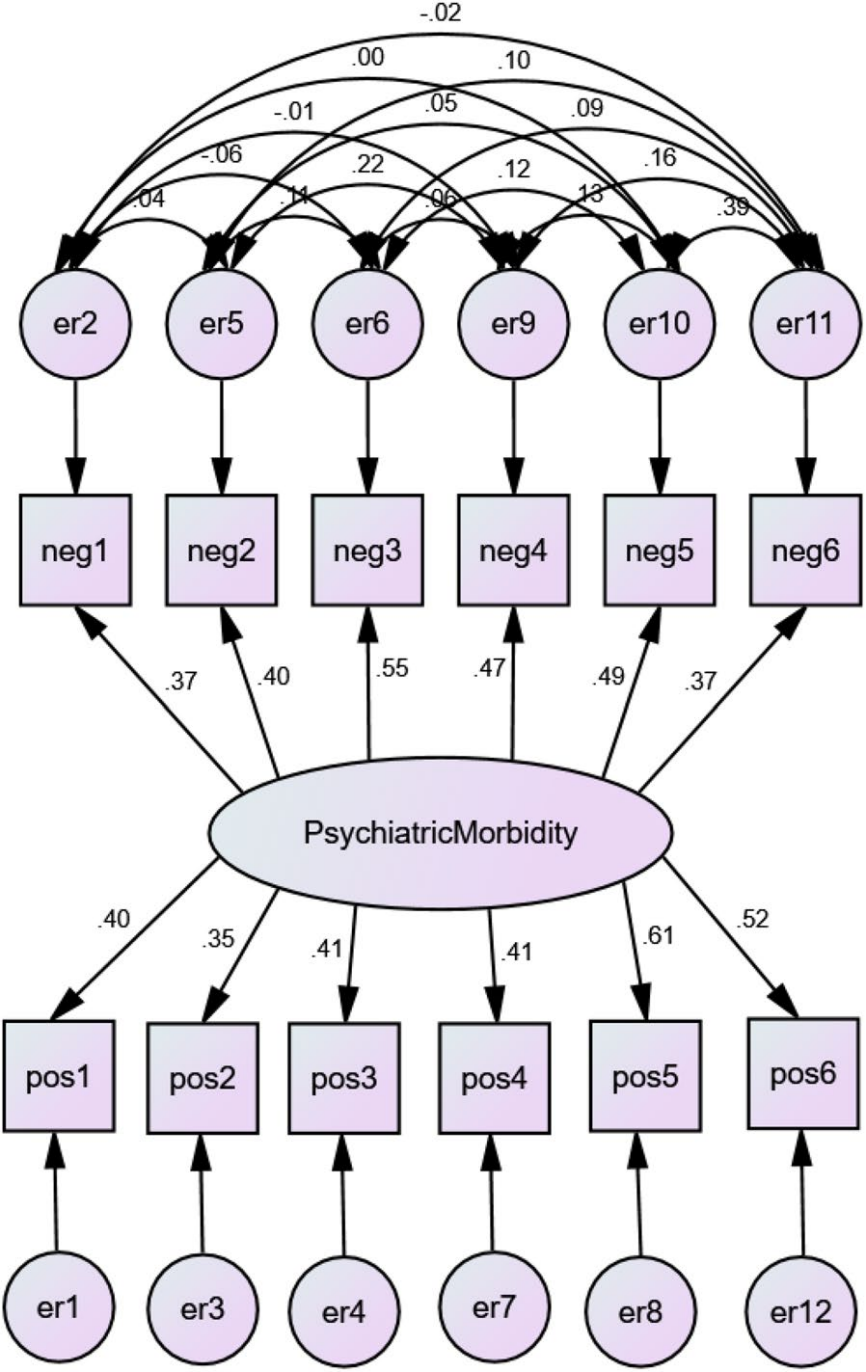
Confirmatory Factor Analysis of one-dimensional model with correlated error terms

Maximum Likelihood Estimation was used to estimate the fit of the four models (Table 7). None of the models are considered good fitting models based on the Normed Fit Index and Comparative Fit Index (Figs. 1, 2, 3, 4), as none of them exceed 0.95 or 0.93 respectively [52].

**Table 7:** The factor structure of the twelve-item General Health Questionnaire (GHQ-12)

Model	Χ^2^	df	p	Χ^2^/df	NFI	CFI	RMSEA (90% CL)	ECVI (90% CL)
1. Three-dimensional	249.10	51	0.000	4.88	0.821	0.849	0.076	0.485
2. One-dimensional	322.78	54	0.000	5.98	0.785	0.795	0.086	0.585
3. Two-dimensional	298.90	53	0.000	5.64	0.785	0.813	0.083	0.552
4. One-dimensional with correlated error terms	212.60	39	0.000	5.45	0.847	0.868	0.081	0.466

*NFI* Normed Fit Index, *CFI* Comparative Fit Index, *RMSEA* Root Mean Square Error of Approximation, *ECVI* Expected Cross Validation Index

Based on the Root Mean Square Error of Approximation (RMSEA), Model 1 was found to be an acceptable fit, while based on the Expected Cross-Validation Index (ECVI), Model 4 is an acceptable fit. Considering all goodness of fit indices, Model 4 was found to be the best of all the options.

Model 1: The three-factor model indicated by the EFA was further examined by CFA below.

Model 2: The one-dimensional model according to the theoretical underpinning of the GHQ-12 was examined by CFA below.

Model 3: The two-dimensional model previously found in the Indonesian version with Likert scoring [38].

Model 4: The one-dimensional model with correlated errors [44].

### Validity coefficients and area under the ROC curve

The threshold values, sensitivity, specificity, PPV, NPV, and AUC of the GHQ-12 based on diagnostic groups (at 2-week prevalence) are summarised in Table 8.

**Table 8:** Performance and ROC area of the GHQ-12 (bimodal scoring)

ICD-10 diagnoses	Threshold	SE	SP	PPV	NPV	AUC
Mood disorders	1/2	0.774	0.433	0.104	0.957	0.702
	2/3	0.717	0.634	0.143	0.963	
Mixed anxiety and depression	1/2	0.902	0.474	0.234	0.965	0.725
	2/3	0.686	0.659	0.263	0.922	
Anxiety disorders	1/2	0.805	0.446	0.157	0.947	0.661
	2/3	0.597	0.624	0.172	0.924	
Any diagnosis	1/2	0.824	0.641	0.679	0.789	0.787
	2/3	0.599	0.798	0.732	0.683	

*SE* Sensitivity, *SP* Specificity, *PPV* Positive Predictive Value, *NPV* Negative Predictive Value, *AUC* Area Under Curve

**Fig. 5:**
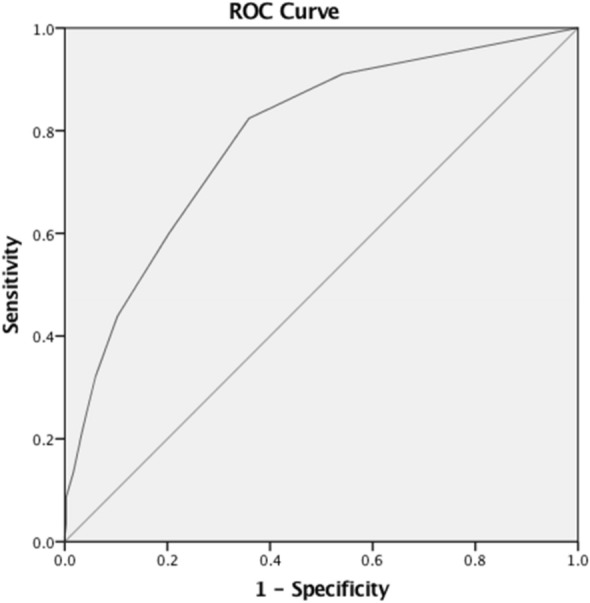
ROC curve of GHQ-12 for ICD-10 psychiatric diagnoses. Bimodal scoring 0-0-1-1

The ROC analysis indicated that the optimal cut-off point for the identification of any diagnosis was 1/2. Sensitivity was 82% while specificity was 64%. The AUC of 0.79 indicates that GHQ-12 is ‘fairly accurate’. The traditional established point system for the AUC specifies that AUC of at least 0.70 is required to ensure fair accuracy [51]. The ROC curve for any ICD-10 diagnosis is presented in Fig. 5. A logistic regression was conducted to predict diagnostic outcome with GHQ-12 screening threshold of 1/2 as a predictor variable. Primary care patients who screened positive based on this threshold have 7.52-fold higher odds of receiving a CIS-R diagnosis (95% CI 3.72–15.20, p < 0.001). Applying this threshold score of ≥ 2 for a further 2 weeks of screening (as part of the recruitment of a trial [9] resulted in the identification of 574 patients who met the screening criteria from 2320 primary care patients screened (24.7%).

## Discussion

The GHQ-12 was found to have good inter-item consistency when used in the Indonesian primary care setting. CFA supports a one-dimensional model with correlated error terms for negatively phrased items which account for response bias. The GHQ-12 is also a ‘fairly accurate’ screening tool with a predictive power for ICD-10 psychiatric diagnosis of nearly 0.8 (AUC = 0.78). The recommended optimal threshold differs depending on the objectives for using the GHQ-12. For use in *Puskesmas*, the goal can be to comprehensively screen for any ICD-10 psychiatric diagnosis even at the risk of a high false positive rate. As such, the optimal threshold for the bimodal scoring is 1/2 points. If the goal is for better discrimination of mood disorders and anxiety disorders [15] it may be more appropriate to adopt the more stringent threshold of 2/3 points.

While for practicality, a more conservative cut-off score will reduce the absolute number of psychiatric interviews to be conducted, one must critically form a decision with the awareness that there are people who would otherwise be diagnosed, who did not meet the screening criteria (False Negatives). Using a cut-off score of 2, the False Negative Rate is 20%, while with a more conservative cut-off score of 3, the False Negative Rate is 31%. If the goal of screening for psychiatric disorders in primary care is to help bridge Treatment Gap, the recommended threshold is 1/2 points, where a score of 2 or above is ‘positive’ for at risk of psychiatric disorders.

The medians of participants with psychiatric diagnosis [4] and those without [1], shows that while the difference of one or two scores may seem trivial, it was sufficient to highlight potential ‘cases’ from other primary care patients. The use of a ‘fairly accurate’ screening tool within clinical setting would facilitate the swift identification of primary care patients at risk of psychiatric morbidity, bolstering the confidence of primary care doctors to conduct in-depth psychiatric interview without fear of making a mistake or offending their patients. Patients who screened positive for indication of mental health problems using this threshold score was found to be 7.52 times more likely to get a diagnosis compared to those who did not screen positive.

The analysis indicates that the Indonesian version of the GHQ-12 may be used to screen for mental health problems among primary care patients. For clinical services, an optimal threshold score for any tool used in screening for mental disorders is necessary to best distinguish at-risk individuals from the remaining population [53]. A screening tool such as the GHQ-12 may have great utility within primary care in Indonesia, particularly as it may have the potential to increase efficiency within an overburdened healthcare system. It could only be introduced, however, if the effective services to support those screened are in place [54], i.e. in primary care clinics which provide mental health services. Those who screened positive should be provided additional information regarding common mental health problems [55]. It could be argued that screening played a key role in identifying patients with indication of mental health problems in the trial we conducted in Indonesia, at very little additional costs to the health systems as screening was embedded into routine procedure [9]. With service expansion planned to reach all 10,000 primary care clinics, policy makers should consider encouraging screening for mental health problems to help clinicians quickly identify patients at risk. Screening, coupled with increased mental health literacy could facilitate the early identification and intervention of mental disorders, which would help bridge Indonesia’s enormous Treatment Gap.

This study’s strength lies in its validation of the utility of the GHQ-12 in Indonesia’s primary care setting, however, it is not without its limitations. While this study confirms the efficacy of the Indonesian version of the GHQ-12 for the Indonesian primary care population, it is not necessarily generalisable for whole populations for general screening, as our sample is limited to primary care attendees. Another limitation is the wide range of mental health disorders captured by the CIS_R and the relatively small number of patients which fall into each of the category (Table 3). This makes it impossible to ascertain if the GHQ-12 was better for screening a specific type of disorder compared to others. Additionally, test–retest reliability was not assessed, further limiting the generalisability of the results. It should be noted that although the GHQ-12 identifies at-risk individuals, to establish an ICD-10 diagnosis requires a full psychiatric interview with qualified clinicians. Further research into the utility of the GHQ-12 in accurately screening for mental disorders among the non-primary care population should be attempted.

The length of waiting time means more patients who agreed to take part in the study left before completing the standardised psychiatric interviews, due to other commitments such as work. This is reflected in the smaller number of men participating in the study (n = 224) compared to women (n = 452). Women have been shown to be more willing to access mental health services than men [56, 57].

If screening were to be implemented across primary care clinics in Indonesia, it is possible its impact would be viewed with concern. Understandably, in clinics with significantly less resources, manpower is limited. Increased consultation time, increased waiting time, and possibly increased working hours for clinicians are but some of the issues anticipated, which might affect the acceptability of screening. As this study took place in real life settings, we observed that medical consultations, including the standardised psychiatric interview, took between 20 to 60 min longer depending on the complexity and severity of symptoms to be addressed. At some clinics, patients meeting the screening criteria were asked to wait for all other patients to have their consultations, drawing strong criticisms from patients who had to wait hours for their consultations. In other clinics, one GP on duty was assigned to handle all patients requiring a psychiatric interview, while all other patients had consultations with other GPs–a seemingly more realistic pathway.

## Conclusions

This study indicates that the Indonesian version of the GHQ-12 is feasible for use as a screening tool for mental health problems among primary care patients. The benefits of screening for mental disorders in primary care must be weighed against other practical considerations. Nonetheless, in Indonesia, where the Treatment Gap for mental disorders is above 95% [3], the benefits could potentially outweigh the additional burden on the health system.

## Data Availability

The dataset supporting the conclusions of this article is available in the University of Cambridge Research Data Repository, at 10.17863/CAM.43399.

## Abbreviations

AUC: Area under the curve
CFA: Confirmatory factor analysis
CIS-R: Clinical Interview Schedule (Revised)
ECVI: Expected Cross-Validation Index
GHQ-12: General Health Questionnaire (12-items)
GP: General practitioner
ICD-10: International Classification of Diseases (10th Edition)
LMIC: Low- and Middle-Income Countries
NPV: Negative predictive value
PPV: Positive predictive value
RMSEA: Root mean square error of approximation
WHO: World Health Organization
